# Broad and Fine Scale Variability in Bacterial Diversity and Cyanotoxin Quotas in Benthic Cyanobacterial Mats

**DOI:** 10.3389/fmicb.2020.00129

**Published:** 2020-02-06

**Authors:** Georgia Thomson-Laing, Jonathan Puddick, Olivier Laroche, Samantha Fulton, Konstanze Steiner, Mark W. Heath, Susanna A. Wood

**Affiliations:** ^1^Cawthron Institute, Nelson, New Zealand; ^2^Department of Oceanography, School of Ocean and Earth Science and Technology, University of Hawai‘i at Mānoa, Honolulu, HI, United States; ^3^Greater Wellington Reginal Council, Wellington, New Zealand

**Keywords:** cyanobacteria, *Phormidium*, *Microcoleus*, biofilm, high-throughput sequencing, 16S rRNA metabarcoding, droplet digital PCR, liquid chromatography-mass spectrometry

## Abstract

Benthic proliferations of *Microcoleus autumnalis* (basionym *Phormidium autumnale*) and closely related taxa are being reported with increasing frequency in streams and rivers worldwide. This species commonly produces the potent neurotoxin anatoxin, and exposure to this has resulted in animal fatalities and human health concerns. Bacterial communities within cyanobacterial assemblages can facilitate processes such as nutrient cycling and are posited to influence cyanobacterial growth and function. However, there is limited knowledge on spatial variability of bacterial communities associated with benthic cyanobacteria and anatoxin content and quotas. In this study, *M. autumnalis*-dominated mat samples were collected from six sites in two New Zealand streams. Associated bacterial communities were characterized using 16S rRNA metabarcoding, anatoxin content by liquid chromatography-mass spectrometry and *anaC* copies using droplet digital PCR. Bacterial assemblages differed significantly when amplicon sequence variants were compared between streams and most sites within streams. These differences were associated with conductivity, DRP, DIN, temperature, anatoxin concentration, and quota. Despite the differences in bacterial community composition; at phyla, class and order levels there was high similarity across spatial scales, with Bacteroidetes (ca. 67%) and Proteobacteria (ca. 25%) dominant. There was significant variability in total anatoxin concentrations between sites in both streams (*p* < 0.001). When the data were converted to anatoxin quotas variability was reduced, suggesting that the relative abundance of toxic genotypes is a key driver of total anatoxin concentrations in mats. This study demonstrates the complexity of microbial communities within *M. autumnalis*-dominated mats and highlights their likely important role in within-mat nutrient cycling processes.

## Introduction

An increasing number of cyanobacterial species are now known to produce cyanotoxins (Mowe et al., 2015; Cirés et al., 2017). Their proliferation, especially in freshwater environments, threatens both water quality and human and animal health (Codd et al., 1999; Codd, 2000; Paerl et al., 2001; Lee et al., 2017; Huisman et al., 2018). The intensification of anthropogenic impacts, as well as changing climates (O’Neil et al., 2012; Paerl and Paul, 2012; Huisman et al., 2018) has led to an increased occurrence of cyanobacterial blooms in both lentic and lotic environments. Research on harmful planktonic cyanobacterial blooms (primarily in lakes) has been undertaken for decades (e.g., Chorus and Bartram, 1999). Whilst toxic benthic cyanobacteria in streams and lakes pose similar issues, research in this area has only recently intensified (Quiblier et al., 2013).

Recent research on toxic benthic cyanobacteria has shown the presence of many cyanotoxins in benthic cyanobacterial mats worldwide (reviewed by Quiblier et al., 2013). For example, microcystins have been identified in benthic samples from Spanish reservoirs (Aboal and Puig, 2005), alpine lakes in Switzerland (Mez et al., 1997), the Nile River and irrigation canals in Egypt (Mohamed et al., 2006), Brazilian streams and water supply reservoirs (Borges et al., 2015) and Californian streams (Fetscher et al., 2015). Other toxins including lyngbyatoxin, saxitoxins, cylindrospermopsin, and nodularin have also been identified in freshwater stream biofilms (Seifert et al., 2007; Borges et al., 2015; Fetscher et al., 2015; Foss et al., 2017, 2018). The most widely reported toxins from benthic mats are anatoxins, with production commonly attributed to *Microcoleus* or *Phormidium*. For example, anatoxins have been reported in mats from streams in France (in 2003; Gugger et al., 2005), New Zealand (in 2005; Wood et al., 2007), Netherlands (in 2011; Faassen et al., 2012), and in California, United States (2013–2015; Bouma-Gregson et al., 2018a). In these cases, research was undertaken in response to rapid dog deaths following consumption of toxic benthic cyanobacteria. Although there has been an increase in the identification of toxin-producing benthic cyanobacteria and their potential risks, most studies remain descriptive, i.e., describing toxin detection and causative species.

In New Zealand rivers and streams the most prevalent benthic cyanobacteria is the filamentous *Microcoleus autumnalis* (basionym *Phormidium autumnale*), which commonly produces anatoxins. There has been an increase in reports of *M. autumnalis*-dominated proliferations (defined in New Zealand as greater than 20% mat coverage of stream bed substrate) in recent years (McAllister et al., 2016). Although dominated by *M. autumnalis*, these mats usually contain many other organisms (e.g., bacteria, diatoms and eukaryotic algae) and inorganic matter (e.g., sediment), bound together by extracellular polymeric substances (Hart et al., 2013; Brasell et al., 2015; Wood et al., 2015). Consortial interactions between microbial communities, specifically heterotrophic bacteria and cyanobacteria are believed to alleviate metabolic constraints through the exchange of carbon, specific growth factors (e.g., vitamins) and limiting nutrients (Paerl et al., 2000, 2001). Molecular approaches have now been extensively used for assessing the diversity of heterotrophic prokaryotes associated with cyanobacteria, with high throughput sequencing-based approaches now commonplace (Cai et al., 2014; Louati et al., 2015; Woodhouse et al., 2015; Berry et al., 2017; Parulekar et al., 2017; Su et al., 2017). To-date most studies have focused on planktonic cyanobacteria, with studies showing that cyanobacterial blooms disturb the natural bacterial community diversity and composition, but that the community returns relatively quickly after the blooms dissipate. Berry et al. (2017) found that *Microcystis* and *Synechococcus* bloom events in Lake Erie coincided with large shifts in non-cyanobacterial community composition that could also be predicted by environmental parameters such as pH, chlorophyll-*a*, temperature, and water movements. Some taxa (i.e., *Actinobacteria*) exhibited more pronounced changes and sensitivity to these shifts in bloom dynamics.

Three recent studies have investigated relationships between *M. autumnalis*-dominated mats and associated microbial communities. Brasell et al. (2015) studied shifts in microbial communities through successional cycles of *M. autumnalis-*dominated mats and showed three distinct phases. Echenique-Subiabre et al. (2018) identified similar bacterial communities in *Microcoleus-*dominated mats from both New Zealand and French streams, with Bray-Curtis dissimilarities between French and New Zealand bacterial communities at the same order of magnitude of those found between the New Zealand streams, suggesting community composition was highly influenced by micro-environmental conditions within the mats. They also identified a strong and significant distance decay relationship (defined as an increasing dissimilarity in community composition with increasing geographic distance) between bacterial communities from New Zealand streams. However, the New Zealand samples were only taken at one site in each stream limiting knowledge on within-stream variability. Bouma-Gregson et al. (2018b) found an association with bacterial community composition within *Microcoleus*-dominated mats from Eel River (California, United States) and presence of *Microcoleus* strains capable of producing anatoxin-a (ATX). The authors also showed that many of the bacteria within the mats had metabolic capacities, such as oxygenic and anoxygenic photosynthesis, carbon respiration, sulfur compound oxidation and urea breakdown, which may benefit *Microcoleus* through the internal cycling of nutrients such as organic nitrogen.

The overarching goal of this study was to investigate the bacterial community assemblages and anatoxin variability in *M. autumnalis*-dominated mats collected across different spatial scales. Five samples were collected from six sites of two streams in New Zealand and a suite of physical and chemical parameters were sampled or measured at each site. Bacterial communities were characterized using 16S rRNA metabarcoding and anatoxin content was determined using liquid chromatography-mass spectrometry (LC-MS). Droplet digital polymerase chain reaction (PCR) was used to determine *anaC* copies number which allowed anatoxin quotas (amount of anatoxin per cell) to be determined. We explored three questions; (1) how does the structure of bacterial communities in *M. autumnalis*-dominated mats vary within sites, within streams and between streams, (2) are anatoxin quotas as spatially variable as total anatoxin content in *M. autumnalis*-dominated mats across varying spatial scales, and (3) are there relationships between the structure of associated bacterial communities, anatoxin content and quota and stream water column physicochemical factors.

## Materials and Methods

### Sampling Sites

**FIGURE 1:**
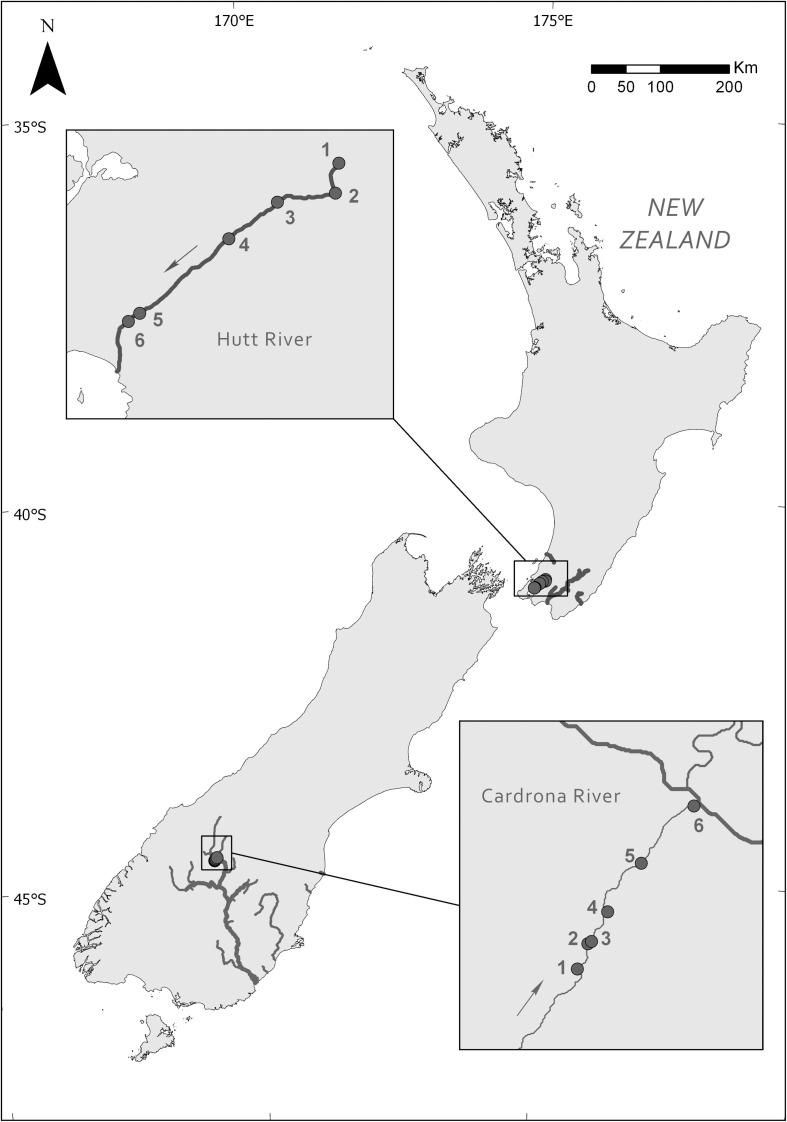
Locations of the six study sites along the Hutt River (Wellington) and Cardrona River (Otago), New Zealand. Arrows indicate the direction of river flow.

Site surveys and *M. autumnalis*-dominated mat sample collection were undertaken at six sites (for geographic coordinates see Supplementary Material 1) along a 23 km longitudinal gradient of the Hutt River (Wellington, New Zealand) on November 28, 2017 and six sites over 9.5 km in the lower Cardrona River (Otago, New Zealand) on April 10–11, 2017 (Figure 1).

The Hutt River is a 56-km long aggrading stream (a stream that is actively elevating its bed by sediment deposition) with a catchment area of 655 km^2^. The upper reaches of the Hutt River catchment are principally native forest and scrublands. Land use in the lower reaches are comprised of low-intensity agricultural, commercial and residential areas.

The Cardrona River has a total catchment area of 337 km^2^, it flows 40 km starting in the steep Cardrona Valley before, draining into the Clutha River. The Cardrona catchment primarily consists of agricultural grasslands ranging from tussock grasslands to high producing pasture grasslands in the upper and lower catchment, respectively. There is a heavy demand for water abstraction in the lower Cardrona River catchment, contributing to the lower stream drying in most years during summer. Upstream of site 2, the Cardrona River goes through a losing reach in which surface water is lost to groundwater with the stream only emerging ca. 400 m upstream of the sampling site. Approximately 1 km downstream the stream goes through another losing reach and was completely dry until emerging from the gravel approximately 200 m upstream of site 4. Site 1 and 6 were on small side stems of the main stream.

### Site Surveys and Sample Collection

At each site, percentage coverage of *M. autumnalis*-dominated mats was visually assessed in five fields of view, equal distances apart, along three transects perpendicular to the bank edge using a bathyscope (Model 0800, Nuova Rade, Italy). At randomly chosen points along the transects, a small section (ca. 2 cm diameter) of a *M. autumnalis*-dominated mat was carefully removed using sterile tweezers, placed in a sterile tube (2 mL), and frozen (−20°C) for later anatoxin and DNA extractions. All mats were well developed and in their expansion phase (McAllister et al., 2016). This resulted in a total of five samples per site for all sites except for Hutt River site 4 (*n* = 4). A small subsample (0.5 g wet weight) of mat from each site was preserved in Lugol’s and water (5 mL). In the laboratory an inverted Olympus CKX-41 was used to confirm *M. autumnalis*-was the dominant cyanobacteria in the samples.

Dissolved oxygen, conductivity (25°C reference temperature), pH and temperatures were measured at each site in the middle of the streams using a handheld water quality sonde (YSI Pro Plus, YSI Inc., Yellow Springs, OH, United States). The water velocity at each site was measured using a hand-held velocity meter (Marsh-McBirney, HACH, Loveland, CO, United States).

Water samples (ca. 0.5–1 L) were collected adjacent to the most downstream mat at each site, syringe-filtered (Whatman GF/C, Whatman, Maidstone United Kingdom; ca. 1.6 μm pore size) and the filtrate frozen (−20°C) for dissolved nutrient analysis. The concentrations of nitrate (NO_3_-N), nitrite (NO_2_-N), ammoniacal-N (NH_4_-N) and dissolved reactive phosphorous (DRP) were determined at Hill Laboratories (Hamilton, New Zealand). Reporting limits for nutrient analyses were 0.001 mg L^–1^ (DRP, NO_3_-N, NO_2_-N, NH_4_-N) for Hutt River samples and 0.002 (NO_2_-N, NO_3_-N), 0.01 (NH_4_-N), and 0.004 mg L^–1^ (DRP) for the Cardrona River samples. Analysis was undertaken on a Lachat Quikchem^®^ flow injection analyzer (FIA + 8000 Series, Zellweger Analytics, Inc.) using APHA (2012) 4500 methods. Dissolved inorganic nitrogen (DIN) was determined by summing NO_3_-N, NO_2_-N, and NH_4_-N.

#### Anatoxin Extraction and Analysis

Frozen *M. autumnalis* mat samples were defrosted, transferred to 20 mL glass vials and lyophilized (Gamma 1-16 LSC freeze-drier; Martin Christ, Germany). Lyophilized material was ground to a fine powder with a sterile metal spatula and two aliquots (for anatoxin and DNA extraction) were weighed (ca. 10–30 mg).

The aliquots for anatoxin extraction were suspended in 1 mL of 0.1% formic acid (made up in Milli-Q water; Merck, Kenilworth, NJ, United States). The samples were vortexed (30 s), frozen (−20°C) and thawed in a sonicator bath (30 min). This was repeated two more times before the anatoxin extracts were centrifuged (12,000 × *g*, 5 min) and diluted 1/20 in 0.1% formic acid and frozen (−20°C). The supernatants were analyzed for dihydroanatoxin-a (dhATX), dihydrohomoanatoxin-a (dhHTX), ATX, and homoanatoxin-a (HTX) using LC-MS/MS as described in Wood et al. (2017b). Anatoxin concentrations were determined using an external standard curve constructed using dilutions of a certified reference material for ATX (National Research Council, Canada; 0.5–20 ng mL^–1^ in 0.1% formic acid). The ATX calibration curve was used to quantify HTX, dhATX, and dhHATX, using a relative response factor of 1. Data were converted to μg g^–1^ (equivalent to mg kg^–1^) by dividing the dilution-adjusted LC-MS results (in ng mL^–1^) by the weight of lyophilized starting material (in mg). Anatoxin quotas (in pg cell^–1^) were calculated by dividing the anatoxin concentration (in μg g^–1^; the sum of the four anatoxin congeners) by the concentration of *anaC* gene copies (in copies g^–1^; see below) and multiplying by 1,000 (to convert μg cell^–1^ to pg cell^–1^).

#### DNA Extraction and *anaC* Gene Quantification

The DNA aliquot was weighed directly into the first tube of the DNeasy PowerSoil^®^ DNA Isolation Kit (QIAGEN, United States) and the extraction was performed following the manufacturer’s protocol. The quantity (>0.5 ng μL^–1^) of the extracted DNA was measured using a NanoPhotometer (Implen, Germany).

Absolute concentrations of the *anaC* gene were measured using a BioRad QX200 Droplet Digital PCR system and *M. autumnalis* specific primers and a probe (Phor-AnaC-F5 5′-ACTAACCGAATCACTTCCACTT-3′, Phor-AnaC-R5 5′-CTC ACCCACCTCACCTTTAG-3′, Phor-AnaC-P5 5′-TTCAGTATT AGCGCAGGCTTTGCC-3′; Kelly et al., 2018). The hydrolysis probe was dual-labeled with a 5′ 6-carboxyfluorescein (6-FAM) fluorescent tag and a 3′ Black Hole Quencher. Each ddPCR reaction included 450 nM of each primer and probe, 1 × BioRad ddPCR Supermix for probes, 1 μL DNA, and sterile water for a total reaction volume of 22 μL. The BioRad QX200 droplet generator partitioned each reaction mixture into nanodroplets by combining 20 μL of the reaction mixture with 70 μL of BioRad droplet oil. After processing, a nanodroplet volume of 40 μL was transferred to a PCR plate for amplification using the following cycling protocol; hold at 95°C for 10 min, 40 cycles of 94°C for 30 s, 60°C 1 min, and a final enzyme deactivation step at 98°C for 10 min. The plate was then analyzed on the QX200 instrument (BioRad). For each ddPCR plate, at least one negative control (RNA/DNA-free water Life Technologies) and one positive control (genomic DNA extracted from a sample known to contain anatoxin) were included. When inhibition was observed, or samples were too concentrated, these were diluted with RNA/DNA-free water (UltraPure^TM^, Life Technologies, CA, United States) and reanalyzed. The results were then converted to copies g^–1^ using the following formula; number of copies per μL × 22 μL (the initial volume of the PCR reaction) × 100 μL (the volume used to elute the DNA during extraction)/weight of lyophilized starting material (in g).

### Bacterial Community Metabarcoding

#### Polymerase Chain Reaction and High-Throughput Sequencing

Amplification of PCR products for high-throughput sequencing was performed using bacterial specific primers to amplify a ca. 400 base pair (bp) sequence in the V3–V4 hypervariable region of the 16S rRNA gene; forward S-D-Bact-0341-b-S-17: 5′-CCT ACG GGN GGC WGC AG-3′ and reverse S-D-Bact-0785-a-A-21: 5′-GAC TAC HVG GGT ATC TAA TCC-3′ (Herlemann et al., 2011; Klindworth et al., 2013) modified to include Illumina^TM^ adapters. Each 50 μL PCR reaction contained 25 μL of AmpliTaq Gold^®^ 360 master mix (Life Technologies, Camarillo, CA, United States), 5 μL of GC enhancer, 2 μL of each forward and reverse primers (10 μM), 15 μL of RNA/DNA free water (Thermo Fisher Scientific, United States) and 1 μL of template DNA. The PCR thermal cycling conditions involved denaturation for 10 min at 95°C, 27 cycles of denaturation at 95°C for 30 s, annealing at 50°C for 30 s, 72°C for 45 s and a final extension at 72°C for 7 min. PCR products were visualized by electrophoresis on 1.5% agarose gel with Red Safe DNA Loading Dye (iNtRON Biotechnology Inc., Kyungki-Do, South Korea) to confirm that only one PCR product was amplified at c. 400 bp. PCR products were cleaned (Agencourt^®^ AMPure^®^ XP Kit; Beckman Coulter, CA, United States), quantified (Qubit^®^ 20 Fluorometer, Invitrogen), diluted to 10 ng μL^–1^ and submitted to New Zealand Genomics Limited (University of Auckland) for high-throughput Illumina sequencing.

#### Bioinformatics

Bioinformatics analysis was performed in QIIME2 (Caporaso et al., 2010). Primers were first removed from sequences, and sequencing data denoised and quality filtered with the default values of the DADA2 algorithm (Version 2018.2.6; Callahan et al., 2016). Amplicon sequence variants (ASVs) were taxonomically assigned with the q2-feature-classifier of QIIME2 and the SILVA reference database (Version 132; Yilmaz et al., 2014). Reference sequences were extracted and trimmed using the aforementioned primers. This was followed by training a multinomial Naïve Bayes classifier on the trimmed reference database and classifying representative sequences using the default values of QIIME2 feature-classifier. The raw FASTQ sequence data has been deposited in the Short Read Archive (NCBI project number PRJNA578643).

### Data Analysis

Anatoxin quota, *anaC* copy number and total anatoxin concentrations were log-transformed prior to analysis in order to normalize their data distributions. One-way analyses of variance (ANOVAs) with Tukey Honest Significant Differences (HSD) pairwise comparisons were used to compare anatoxin concentrations, anatoxin quota, and *anaC* copy numbers between sites along either the Hutt or Cardrona rivers. Sites were assumed to be independent for all analyses. Welch Two Sample *t*-tests were used to compare toxin variables between the Hutt and Cardrona rivers. Statistical analyses were conducted using R software (R Studio Team, 2015; R Core Team, 2016) with ggplot2 (Wickham, 2016) and heplot (Fox et al., 2018).

Prior to all analyses on bacterial community data, unassigned sequences, chloroplast, mitochondrial and cyanobacterial reads were removed from sequence data. ASVs present in method controls (*n* = 30) were removed from the dataset. Percentage stacked bar graphs were used to visualize the abundance of taxa in each sample using data prior to rarefaction. To assure adequate sequencing depth and normalize data information among samples, rarefaction curves were produced with the vegan package (Oksanen et al., 2018) using R software (Supplementary Material 2; R Studio Team, 2015; R Core Team, 2016). For alpha and beta diversity analysis sequencing depth was rarefied at 2,500 reads per sample through random subsampling with no replacement. Three samples with <2,500 reads were removed during rarefaction and excluded from further analysis (Hutt River 2B, 3E, and Cardrona River 4C).

Beta-diversity assessment between streams was performed by transforming the rarefied data from both streams with a square root and Wisconsin standardization, followed by the production of a Bray-Curtis dissimilarity matrix. Based on this matrix, a non-metric multidimensional scaling (NMDS) was created to visualize the ordination of samples. Beta-diversity assessment (as previously described) was also carried out separately on Hutt River and Cardrona River data to determine finer-scale patterns. Statistical analyses were conducted using R software (R Studio Team, 2015; R Core Team, 2016) with ggplot2 (Wickham, 2016), phyloseq (McMurdie and Holmes, 2013), and vegan (Oksanen et al., 2018) packages.

The multivariate differences in bacterial community composition between the two streams as well as between the sites in each stream was assessed using distance-based permutational analysis (PERMANOVA; Anderson et al., 2008) followed by pairwise tests. Analyses were based on Bray-Curtis similarities using square-root transformed data that had been rarefied as described above. This was conducted using PRIMER 7 with PERMANOVA + add-on (PRIMER-E Ltd., Plymouth, United Kingdom).

The envfit function (vegan package) was used to assess the correlation between the ordination of the bacterial communities and log-transformed environmental variables as well as anatoxin concentration and quota. Significant variables (*p* < 0.05) were overlaid on the nMDS (described above) as vectors with the length of arrow proportional to the correlation strength and the arrow direction indicating the relationship between bacterial community and environmental variable.

## Results

### *Microcoleus autumnalis*-Dominated Mat Cover

*Microcoleus autumnalis*-dominated mat cover was greater than 20% at all sites except for Hutt River site 3, where cover was 5% and Cardrona site 6, where cover was 10%. The highest *M. autumnalis-*dominated mat cover in both streams, 75 and 80%, was recorded for Hutt River sites 1 and 2, and Cardrona River site 4, respectively (Supplementary Material 3).

### Environmental Factors

Conductivity varied between all sites, with the lowest conductivity in the Hutt River measured at site 2 (96 μS cm^–1^) and the highest at site 5 (116 μS cm^–1^). In the Cardrona River, conductivity tended to increase from the upstream to downstream sites, from 95 μS cm^–1^ at site 1 and 131 μS cm^–1^ at site 6 (Supplementary Material 3).

DIN concentrations in the Hutt River were higher at the lower three sites, specifically site 4 where DIN measured 0.19 mg L^–1^. Upstream Hutt River sites tended to have less DIN, with the lowest concentrations measured at site 2 and 3 (0.097 and 0.071 mg L^–1^). There was more variability in DIN concentrations in the Cardrona River, with lower concentrations (sites 2 and 3; 0.056 mg L^–1^) and much higher concentrations, 0.36 and 0.42 mg L^–1^ recorded at sites 4 and 5, respectively (Supplementary Material 3).

In the Hutt River, water column DRP tended to decrease from upstream (c. 0.025 mg L^–1^) to the most downstream site (site 6; <0.001 mg L^–1^). Except for Cardrona River site 6, where DRP was 0.015 mg L^–1^, DRP was generally below <0.01 mg L^–1^ (Supplementary Material 3).

In the Hutt River, water temperature increased from upstream to downstream sites; (21.1 and 23.6°C at sites 1 and 6, respectively), except for site 3 where temperature was 20.8°C. Cardona River had substantially lower water temperature (ranging from 14.9 to 11.5°C at sites 3 and 5, respectively; Supplementary Material 3).

Point water velocity ranged from 0.42 m s^–1^ (site 4) to 0.16 m s^–1^ (site 5) at Cardrona River sites. Higher velocities were recorded in the Hutt River, ranging from 0.15 m s^–1^ (site 6) to 0.83 m s^–1^ (site 3; Supplementary Material 3).

### Anatoxin Concentrations, Quotas and Congeners in *Microcoleus autumnalis*-Dominated Mats

**FIGURE 2:**
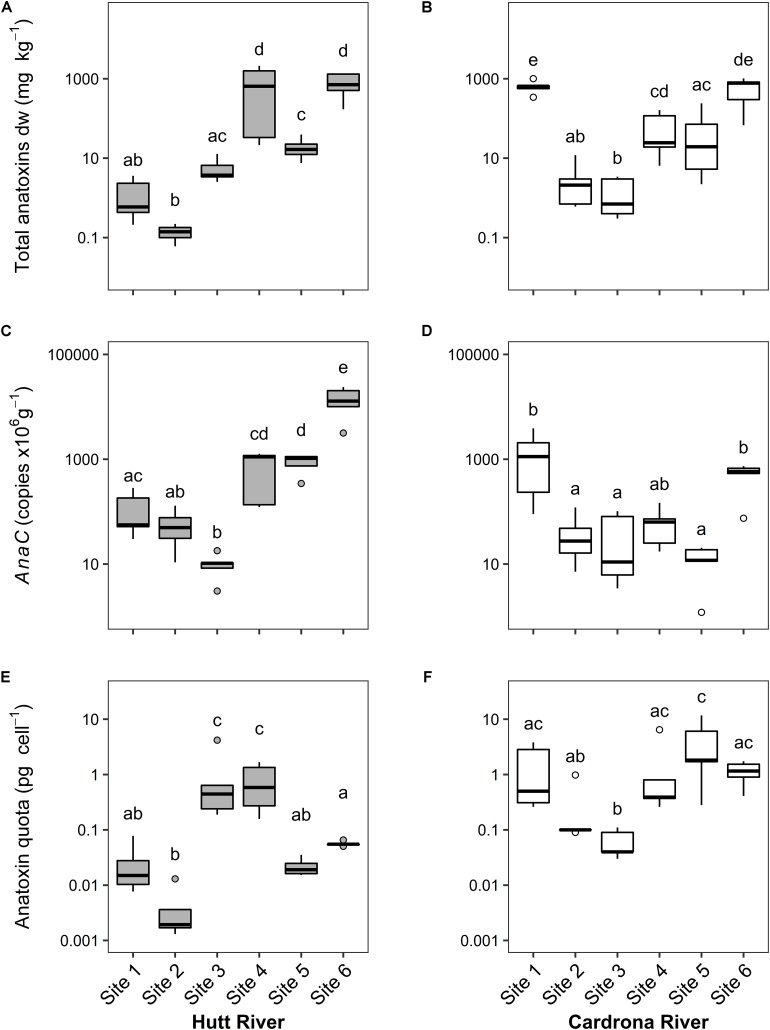
**(A,B)** Total anatoxin (i.e., the sum of the four anatoxin congeners); **(C,D)** concentrations of *anaC* copy numbers; and **(E,F)** anatoxin quotas (amount of toxin per toxic cell) from *Microcoleus autumnalis*-dominated mats collected from sites along the Hutt River (Wellington, New Zealand) or the Cardrona River (Otago, New Zealand). Sites are arranged along *x*-axis in an upstream (left) to downstream (right) gradient. *n* = 5 for all sites except for Hutt River site 4 (*n* = 4). Note that *y*-axes are different log scales. Solid black lines indicate the median, box shows 1st and 3rd quartiles, whiskers extend to the last data point within 1.5 times the inter-quartile range. Open circles are outliers beyond this range. Shared letters indicate no significant difference (*p* > 0.05) between sites (One-way ANOVA, Tukey HSD). Dw, dry weight; pg, picograms.

Median total anatoxins concentrations in the Hutt and Cardrona rivers were 7.56 and 21.95 mg kg^–1^ dw, respectively. Despite this difference in medians, there were no significant differences between total anatoxin concentrations in the Hutt and Cardrona rivers (*t*(54) = 1.17, *p* = 0.24) due to high within-stream and within site variability. In general, median total anatoxin concentration (the sum of the four structural congeners) per site increased from upstream to downstream, in both the Hutt and Cardrona rivers, with Cardrona site 1 being an exception to this trend (Figures 2A,B). There were statistically significant differences in total anatoxin concentrations between sites for both the Hutt River (*F*_5_,_23_ = 39.37, *p* < 0.001) and Cardrona River (*F*_5_,_24_ = 22.08, *p* < 0.001), with the highest median concentrations, 710 mg kg^–1^ dried weight (dw) and 778 mg kg^–1^ dw, in the Hutt and Cardrona rivers, respectively, measured at Hutt site 6 and Cardrona site 6 (Figures 2A,B). Relatively high anatoxin concentrations were also measured at Cardrona site 1 (median: 647 mg kg^–1^ dw). The highest maximum total anatoxin concentrations in each stream were measured in samples from Hutt River site 4 (2,116 mg kg^–1^ dw) and Cardrona River site 6 (1,017 kg^–1^ dw; Figures 2A,B).

Comparison of the *anaC* gene copy numbers between Hutt and Cardrona rivers revealed a significant difference (*t*(55) = -2.24, *p* = 0.03), with higher copy numbers in Hutt River samples (median = 135) in comparison to Cardrona River (median = 68.0). In the Hutt River, *anaC* gene copy numbers differed significantly between sites (*F*_5_,_23_ = 39.72, *p* < 0.001) with downstream sites 4, 5, and 6 having significantly higher (at least 20- and 200-fold higher, respectively) copy numbers than upstream sites (Tukey HSD, *p* < 0.001; Figure 2C). Similarly, in the Cardrona River there was significant variation in *anaC* gene copies between sites (*F*_5_,_24_ = 8.80, *p* < 0.001); however, this trend was not longitudinal with sites 1 and 6 having at least 15- and 10-fold higher copy numbers than other sites, respectively (Figure 2D).

Between the Hutt and Cardrona rivers, there were significant differences in anatoxin quotas (*t*(51) = 4.50, *p* < 0.001), with quotas higher in the Cardrona River (median = 0.40 pg cell^–1^) compared to the Hutt River (median = 0.054 pg cell^–1^). Anatoxins quotas differed significantly among Hutt River sites (*F*_5_,_23_ = 20.76, *p* < 0.001) and Cardrona River sites (*F*_5_,_24_ = 8.12, *p* < 0.001). In the Hutt River, quotas were significantly higher at sites 3 (median 0.44 pg cell^–1^) and site 4 (median 0.58 pg cell^–1^) in comparison to other sites, specifically site 2 (median 0.002 pg cell^–1^; Tukey HSD, *p* < 0.01; Figure 2E). In the Cardrona River, site 5 and 6 had the highest median quotas (1.82 and 1.16 pg cell^–1^, respectively) and the lowest median quota was measured in site 3 samples (0.04 pg cell^–1^). The highest quota values overall were measured in Cardrona River samples (6.45 and 11.72 pg cell^–1^, sites 4 and 5, respectively; Figure 2F).

**FIGURE 3:**
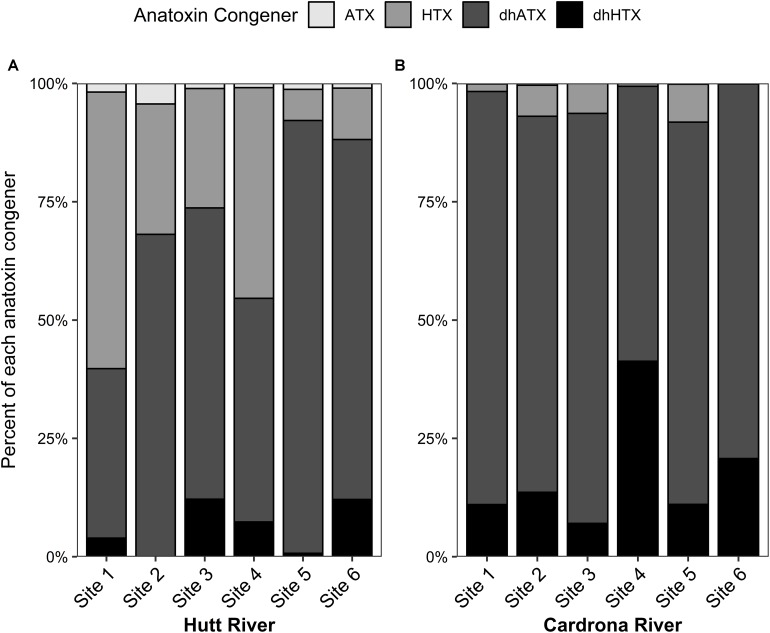
Stacked bar graph showing the average percentage of each anatoxin congener in *Microcoleus autumnalis*-dominated mat samples collected from **(A)** Hutt River (Wellington, New Zealand) and **(B)** Cardrona River (Otago, New Zealand). ATX, anatoxin-a; HTX, homoanatoxin-a; dhATX, dihydroanatoxin-a; dhHTX, dihydrohomoanatoxin-a. *n* = 5 for all sites except for Hutt River site 4 (*n* = 4). Sites are arranged along *x*-axis in an upstream (left) to downstream (right) gradient.

Dihydro-ATX was the most abundant congener detected in the *M. autumnalis*-dominated samples at Cardrona River sites (58–87%) and at all Hutt River sites (>47%) except for site 1, where HTX was most abundant (58%; Figures 3A,B). In the Hutt River, HTX was the second most abundant anatoxin congener at all sites, contributing 6–58% (Figure 3A). In contrast, dhHTX was the second most abundant congener in the Cardrona River, contributing between 7–20% of total toxins at each site (Figure 3B). ATX contributed less than 1% abundance to the congener profiles of samples from the Cardrona and Hutt rivers (Figures 3A,B).

### Bacterial Communities in *Microcoleus autumnalis*-Dominated Mats

Analysis of the 16S rRNA gene sequences showed that all the samples were dominated by cyanobacterial reads (mean ± SD: 76.0 ± 18.3% of the total reads per site) with Phormidiaceae contributing 98.7 ± 2.6% of total cyanobacterial reads. The remaining 1.3% cyanobacterial reads were primarily Pseudanabaenales followed by Leptolyngbyales and to a lesser degree, Synechococcales and Caenarcaniphilales (Melainabacteria).

**FIGURE 4:**
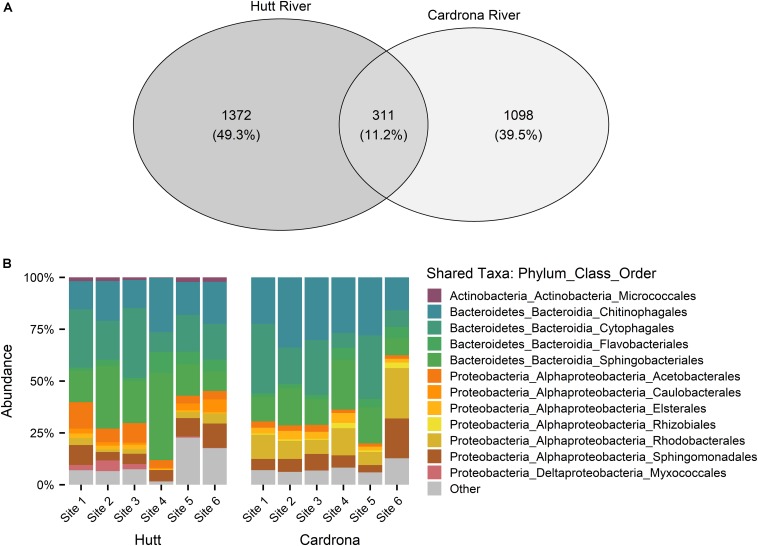
**(A)** Venn diagram of bacterial amplicon sequence variants (ASVs) from *Microcoleus autumnalis*-dominated mats from the Hutt and Cardrona rivers, New Zealand. ASVs are based on 16S rRNA gene fragment sequences. Number of ASVs in each river and shared ASVs between rivers are shown excluding chloroplast and cyanobacterial reads for post rarefaction of sample sizes to 2500 reads per sample. **(B)** Stacked bar graph showing the relative abundance of the shared ASVs from the bacterial 16S rRNA sequences in samples from five Hutt River (Wellington, New Zealand) sites, and six Cardrona River (Otago, New Zealand) sites. The most abundant phyla (contributing >1% of total abundance in each river) are stated. “Other” composed of rare phyla (<1%).

**FIGURE 5:**
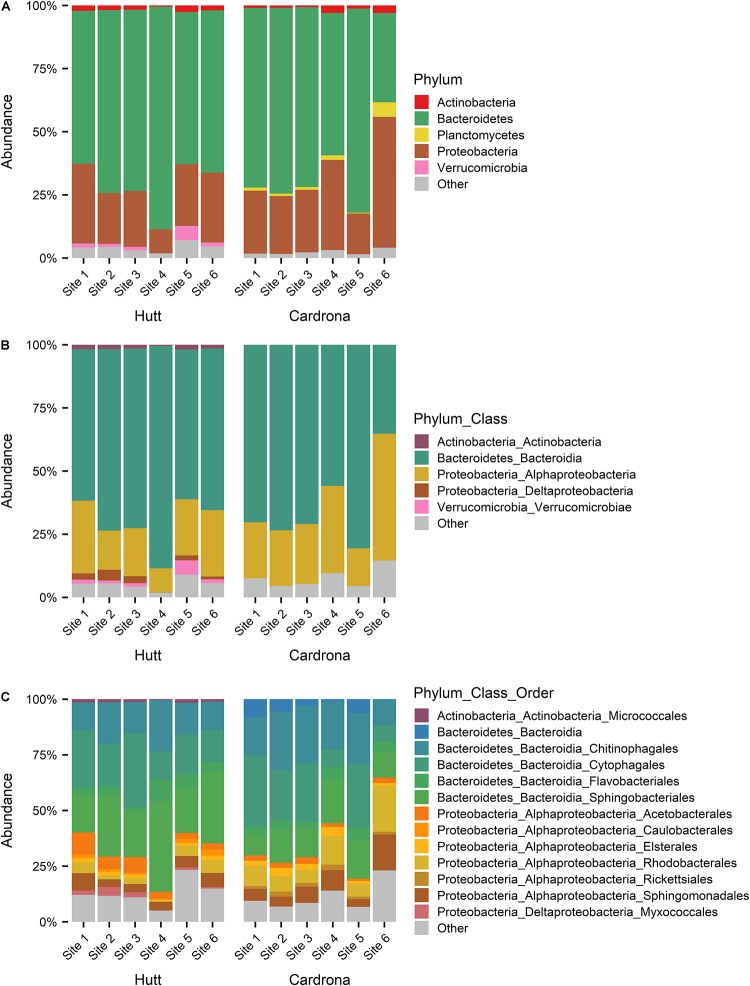
Stacked bar graph showing the relative abundance of bacterial 16S rRNA sequences at **(A)** phyla, **(B)** class, **(C)** order taxonomy levels in *Microcoleus autumnalis*-dominated samples from five Hutt River (Wellington, New Zealand) sites, and six Cardrona River (Otago, New Zealand) sites. Cyanobacterial reads were excluded, and the most abundant phyla (contributing >1% of total abundance in each river) are stated. “Other” composed of rare phyla (<1%). Sites are arranged along *x*-axis in an upstream (left) to downstream (right) gradient.

Following the removal of cyanobacterial sequences and post-rarefaction, a total of 1372 and 1098 ASVs were present across all Hutt and Cardrona river samples, respectively (Figure 4A). In rarefied data, only 11.2% of all ASVs were shared among the Hutt and Cardrona samples with the remaining 88.8% unique to either stream (Figure 4A). Upon examination of the shared ASVs (Figure 4B), patterns in bacterial composition were consistent with those seen when the total bacterial communities were analyzed (Figure 5).

When examining the total bacterial communities in both streams but following the exclusion of all cyanobacterial reads, the most abundant bacterial phyla present was Bacteroidetes, contributing 69.9 ± 11.2% and 64.7 ± 17.8% of the total abundance of the bacterial community in samples from the Hutt and Cardrona rivers, respectively (Figure 5A). Among Bacteroidetes, Bacteroidia dominated the class level, composing 69.4 ± 11.4% (of total abundance) in the Hutt River and 64.4 ± 17.8% in the Cardrona River (Figure 5B). The top three orders of Bacteroidetes in order of abundance in the Hutt River were Sphingobacteriales (26.5 ± 10.1% of total abundance), Cytophagales (20.7 ± 9.9%), and Chitinophagales (15.9 ± 4.9%) with Flavobacteriales present at a lower abundance (4.7 ± 3.2%; (Figure 5C). *Pedobacter* (Sphingobacteriales; Bacteroidetes) was the most abundant genus (20.9 ± 11.6%) in the Hutt River. Similar bacterial orders were dominant in the Cardrona River; however, with some differences in respective abundances with successive dominant orders from Cytophagales (20.9 ± 12.2%), Chitinophagales (20.9 ± 7.6%), and Sphingobacteriales (14.1 ± 5.9%; Figure 5C). Flavobacteriales were present at lower abundance (4.5 ± 2.7%) in the Cardrona River. The most abundant genera in the Cardrona River differed to the Hutt River with *Ferruginibacter* (8.2 ± 4.5%) and *Sediminibacterium* (7.8 ± 5.1%) from the Chitinophagales order dominating.

Proteobacteria was the second most abundant phylum, making up 22.5 ± 8.5% of the Hutt River and 29.4 ± 14.2% of the Cardrona River total bacterial community composition. Alphaproteobacteria was dominant within Proteobacteria, contributing 20.1 ± 8.3% of total abundance in the Hutt River whereas although present, Deltaproteobacteria contributed less (2.1 ± 2%) to the total abundance (Figure 5B). Within Alphaproteobacteria, most of the sequences in the Hutt River samples were classified in the orders of Acetobacterales (5.3 ± 3.2% of total abundance), Sphingomonadales (5.0 ± 2.7%), and Rhodobacterales (3.3 ± 2.3%; Figure 5C). A similar pattern was observed in the Cardrona River with Alphaproteobacteria the dominant Proteobacteria, composing 27.9 ± 14.1% of total abundance (Figure 5C). In the Cardrona, Rhodobacterales and Sphingomonadales were the most abundant Alphaproteobacteria, contributing 10.2 ± 6.6% and 7.6 ± 5.2% of total abundance, respectively.

**FIGURE 6:**
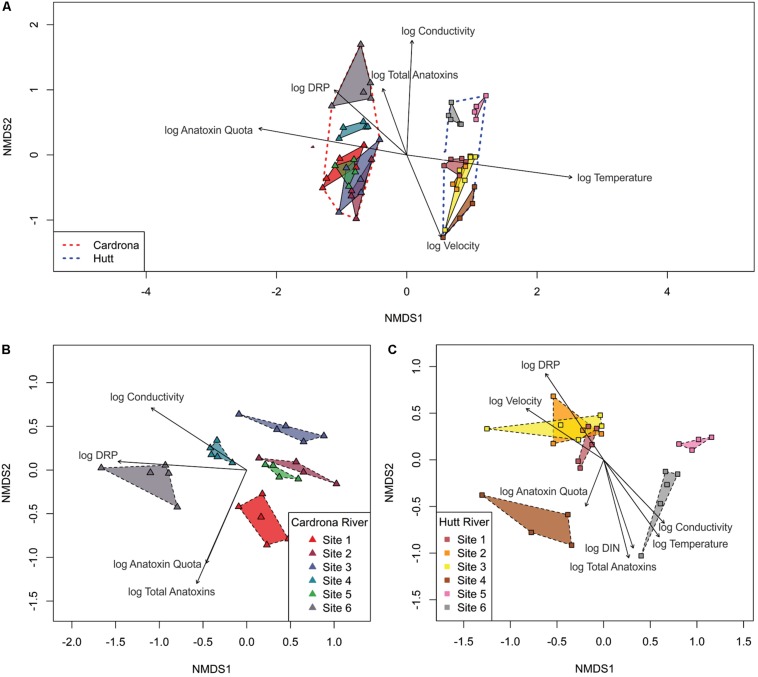
Non-metric, multi-dimensional scaling ordination (confidence level = 0.95) based on Bray-Curtis similarities with 999 permutations of bacterial communities in *Microcoleus autumnalis*-dominated mat samples from; **(A)** the Hutt and Cardrona rivers, **(B)** the Cardrona River, and **(C)** the Hutt River at the individual sequence level. Relationships with dissolved reactive phosphorus (DRP), conductivity, velocity, dissolved inorganic nitrogen (DIN), temperature, and total anatoxin concentrations (log transformed) are indicated by vectors. Only significant relationships (*p* < 0.05) are shown.

Multidimensional scaling based on Bray-Curtis similarities between bacterial communities (using ASVs), showed a clear separation between the two streams (Figure 6A). Analysis of overall bacterial community structure using PERMANOVA revealed a significant difference between the Hutt and Cardrona rivers (pseudo-F = 18.4, *p* < 0.001). Due to this high degree of separation, the relationship between bacterial communities and the environmental variables and anatoxin concentration was also compared for each stream separately (Figures 6B,C).

Bacterial communities from the Hutt River sites 1, 2, and 3 (upstream sites) formed a distinct cluster, separate to the mid-stream site 4 and the downstream sites 5 and 6, which also grouped together (Figure 6C). PERMANOVA analysis showed that the bacterial communities were significantly different between Hutt River sites (pseudo-F = 5.14, *p* < 0.001). Pairwise analysis revealed that all sites were significantly different, with the only exception being sites 2 and 3 (Supplementary Material 4). In the Cardrona River, except for an overlap between two sites (sites 2 and 5), each site clustered distinctly with significant differences between sites (PERMANOVA; pseudo-F = 4.78, *p* < 0.001; Figure 6B). Furthermore, pairwise tests showed that despite this overlap, the bacterial communities at all sites were significantly different from each other (Supplementary Material 5).

#### Relationship Between Water Chemistry, Total Anatoxins and Quota’s and Bacterial Communities

When comparing between streams, the bacterial community composition in the Hutt River was correlated with the higher velocity and temperature (Figure 6A). In comparison, higher DRP, anatoxin quota and concentration were associated with bacterial community composition in the Cardrona River (Figure 6A). In both streams, conductivity was also significantly associated with the distribution of bacterial communities between sites.

The environmental variables that were strongly associated with bacterial community compositions in the Cardrona River were conductivity and DRP, with higher levels correlated with site 4 and 6, respectively (Figure 6B). There was also a relationship between anatoxin concentration/quotas and bacterial communities in the Cardrona River; however, this relationship was not specific to one site.

The composition of clustered bacterial communities identified at the upper sites 1, 2, and 3 in the Hutt River were associated with the higher DRP and water velocity found at these sites in comparison to downstream sites (Figure 6C). Higher DIN, conductivity and temperature were correlated with the bacterial community composition at Hutt River site 6 (most downstream site). Additionally, higher total anatoxin concentrations as well as anatoxin quota were strongly associated with the composition of the bacterial communities at Hutt River sites 4 and 6.

## Discussion

Using 16S rRNA metabarcoding we characterized diverse bacterial assemblages in *M. autumnalis*-dominated mats from two large fluvial systems (the Hutt and Cardrona rivers) in New Zealand. This data, in concert with previous studies (Brasell et al., 2015; Bouma-Gregson et al., 2018b; Echenique-Subiabre et al., 2018) clearly demonstrate that *Microcoleus*-dominated mats are complex microbial communities. Although there were fine-scale differences in bacterial community composition between streams, sites and samples when analyzed at the ASV taxonomic level; at higher taxonomic ranks (i.e., phyla) both the Hutt and Cardrona river bacterial communities were largely composed of Proteobacteria and Bacteroidetes. This is similar to that described by Brasell et al. (2015) who worked on a single site in the Hutt River, and Echenique-Subiabre et al. (2018) who investigated *Microcoleus*-dominated mats in the Tarn River in France and eight New Zealand rivers and streams. In contrast to these studies, the present research showed that Bacteroidetes dominated whereas, both Brasell et al. (2015) and Echenique-Subiabre et al. (2018) identified Proteobacteria as the dominant phylum. Furthermore, both Betaproteobacteria and Alphaproteobacteria were the main and largely equal contributors to Proteobacterial abundance in the studies of Brasell et al. (2015) and Bouma-Gregson et al. (2018b), differing with the almost exclusive presence of Alphaproteobacteria in the present study. As a result of this, Burkholderiales (Betaproteobacteria), although present in samples, did not contribute the bacterial composition to the same degree that was identified by both Brasell et al. (2015) and Echenique-Subiabre et al. (2018). Alphaproteobacteria orders Sphingomonadales and Rhodobacterales were prominent across all studies (Brasell et al., 2015; Echenique-Subiabre et al., 2018). Bouma-Gregson et al. (2018b) worked on *Microcoleus*-dominated mats in the Eel River, California and the abundant phyla described in their manuscript are more aligned to the present study with Bacteroidetes dominant. Differences between studies could also be partly due to biases that are introduced by employing different high-throughput sequencing and bioinformatic methodologies; for example, the selection of DNA metabarcoding region and primers, sequencing platforms, the preparation of sequence libraries including the reference database used for taxonomic assignment and subsequent clustering methods (eg., Balvočiūtė and Huson, 2017; Abdelfattah et al., 2018; Porter and Hajibabaei, 2018; Bukin et al., 2019; Zinger et al., 2019). A limitation of the current study is the low rarefaction threshold, which may reduce the overall coverage of biodiversity compared to what has been identified in other studies.

There were distinct bacterial communities identified at a range of spatial scales with significant differences identified between streams and sites. The between stream differences were apparent despite the same organism, *Microcoleus*, dominating the mat community in both streams. In addition to environmental factors (discussed below) there are several considerations regarding the influence of dominant taxa in the mat assemblage. The two streams were sampled at different times of the year; early summer and late autumn (Hutt and Cardrona rivers, respectively). It is possible the residence period of the mats prior to sample collection differed between each river due to differences in seasonal growth and flushing flows. Brasell et al. (2015) described distinct phases of succession in microbial communities associated with *Microcoleus*, highlighting that mat age is an important consideration that may explain differences between stream assemblages. There is also a potential that *Microcoleus* is genetically different between streams (this study only examined the 16S rRNA gene). Genetic variation in functional genes can result in biological consequences, for example variability in anatoxin concentrations among *Microcoleus*-dominated mats (Wood and Puddick, 2017), that may in turn influence the associated microbial community. Additionally, there may be other co-inhabiting organisms in the mats (i.e., diatoms or other eukaryotic algae) that differ between streams and sites and could influence co-inhabiting bacteria. Similar patterns found by Echenique-Subiabre et al. (2018) showed greater dissimilarity between New Zealand streams, compared to dissimilarity between New Zealand streams and a French stream, which led them to suggest that factors within mat habitats were more likely to regulate associated microbial community in comparison to external factors.

At a finer spatial scale, some sites exhibited considerable within-site variability that was sometimes greater than between-site variability. Additionally, when there was considerable overlap in communities between sites (i.e., Cardrona River sites 2 and 5), these patterns did not consistently follow spatial patterns such as a predicted higher similarity between sites situated next to each other. Stream water potentially provides an inoculum for microbial communities; however, the results of this study indicate this may not be the key inoculum. This was especially apparent in the Cardrona River between sites 2 and 3, which are very close to each other and connected by flowing water but isolated from all other sites (due to the stream being dry, i.e., losing reach). Despite this, the communities in the *M. autumnalis*-dominated mats at these sites were significantly different. Cardrona sites 4, 5, and 6 were all connected by flowing water, but there were significant differences in bacterial community composition between these sites. This suggests other environmental parameters either external or within mats are playing a significant role in driving community structure.

It is well known that environmental conditions can influence the bacterial community composition of stream bacteria (e.g., Fierer et al., 2007; Zeglin, 2015). The data in this study indicates that temperature, water chemistry, concentration of DRP as well as conductivity, and water velocity may play a role in structuring bacterial communities. However, caution is advised when relating water column measurements to within mat communities. Conditions in *M. autumnalis*-dominated mats can be markedly different to those of the overlying stream water. For example, Wood et al. (2015) showed that water within *M. autumnalis*-dominated mats can have up to 320-fold higher DRP concentrations compared to surrounding stream water as a result of desorption of phosphorus bound to sediment within the mat matrix as pH and dissolved oxygen changed markedly over diurnal cycles and were very different to water column values. It is likely that micro-environmental conditions, such as these, have a substantial effect on bacterial community structure, but that these organisms also play an important role in creating and maintaining these conditions. Bouma-Gregson et al. (2018b) suggested, based on metagenomic analyses, that the microbial community contributes to many processes such as phosphorus mineralization in mats through the scavenging of phosphorus using acid phosphatases during periods of pH elevation. Nitrogen cycling within mats, with the degradation of urea as a nitrogenous waste product recycled by microbial organisms, is also suggested to decouple nitrogen requirements from overlying contributions from the external system (Bouma-Gregson et al., 2018b); a state that is posited to occur when mats are mature and established. Further studies, which use techniques that enable conditions within *M. autumnalis*-dominated mats to be characterized are required to fully understand the relationship between within-mat conditions and bacterial community composition. In addition to exploring structural differences among communities, enhanced knowledge on their functions (i.e., metatranscriptomics) would provide valuable insights as to whether communities are involved in similar processes in the mat environments, despite differences in community composition.

Statistical analysis identified a relationship between bacterial community structure in *M. autumnalis*-dominated mats and total anatoxin concentration as well as anatoxin quota. Bouma-Gregson et al. (2018b) also identified a difference between the bacterial communities from *Microcoleus*-dominated mat samples with and without the anatoxin-a gene operon. Although it was possible that bacteria present were influencing selection for toxin-producing cyanobacterial strains, Bouma-Gregson et al. (2018b) considered it more likely that cyanobacterial-produced compounds, such as anatoxin-a, were partly responsible for shaping the microbial community. Harland et al. (2013) and Harland et al. (2014) hypothesized that cyanotoxins may be involved in intra- or inter-species interactions, specifically chemotaxic signaling either between *M. autumnalis* cells and/or other micro-organisms. Their laboratory-based studies showed that up-regulation in anatoxin production occurred early in the growth phase, which could indicate a role for the toxin in the colonization stage of cyanobacterial mats.

The anatoxin concentration data collected in the present study conformed to patterns observed in other studies, with considerable spatial variability between mats at each site and between sites (Wood et al., 2010, 2017a; McAllister et al., 2018). In general, the highest median anatoxin concentration values (ca. 700 mg kg^–1^) for each stream were markedly higher than most of those reported for other New Zealand streams (McAllister et al., 2016). The relative abundance of anatoxin congeners observed in the samples collected during the present study generally contained dhATX as the dominant congener followed by HTX or dhHTX. This was consistent with results from other New Zealand streams (McAllister et al., 2016). This highlights the importance of analyzing for all anatoxin congeners (not solely ATX) when undertaking research on *M. autumnalis* and when assessing the human and animal health risks.

Although numerous studies have shown high variability in anatoxin content of *Microcoleus*-dominated mats, few studies have explored differences in anatoxin quotas (Wood and Puddick, 2017; Kelly et al., 2018). In general, converting the data to anatoxin quotas reduced the variability among the mat samples in comparison to total toxin concentrations. This corroborates the findings of Wood and Puddick (2017) who demonstrated that the abundance of toxic genotypes within *M. autumnalis*-dominated mats is likely to be the main cause of variability in total anatoxin concentrations among mats. Although variability was reduced by the conversion of total toxin to anatoxin quota, some differences remained. These could be due to an up- or down-regulation in anatoxin production caused by biotic or abiotic factors (discussed below), however, given that no consistent patterns were observed this is more likely due to differences in the amount of toxin produced by different strains of toxic *M. autumnalis* cohabitating in the mats. Wood et al. (2012) isolated and cultured 30 strains of *M. autumnalis* from four small sections of benthic mats. Among the toxic strains there were 100-fold differences in the amount of anatoxin produced.

Although environmental factors regulating anatoxin production were not directly investigated in this study, previous research by Heath et al. (2011) suggested that toxin-producing strains in the Hutt River “out-competed” non-toxic *M. autumnalis*-dominated strains at temperatures above 15°C, but subsequent studies in other streams have not observed this pattern (Wood et al., 2017a). Heath et al. (2014) investigated the effects of nitrogen and phosphorus on the growth of two *M. autumnalis* strains (toxic and non-toxic) in a laboratory-based study. Cell concentrations and maximum growth rates were higher for the non-anatoxin-producing strain regardless of treatments, suggesting that specific genotypes may have environmental preferences. Experiments using a larger number of strains are required before definitive conclusions can be drawn about environmental preferences of toxic versus non-toxic *M. autumnalis* genotypes.

## Conclusion

This study continues to highlight the complexity of the microbial communities within *M. autumnalis*-dominated mats. Some of these species will be involved in internal nutrient cycling processes, potentially explaining why establishing relationships between the abundance of *M. autumnalis*-dominated mats and environmental variables measured in stream water is complex or impossible. Further studies investigating the functions associated with these communities are recommended. The results of the present study corroborated previous research and show high variability in total anatoxin concentrations at varying spatial scales. When the data were converted to anatoxin quotas this variability was reduced, suggesting that the relative abundance of toxic genotypes within *Microcoleus*-dominated mats is a key driver of differences in total anatoxin concentrations among mats. Factors that regulate the relative abundance of toxic and non-toxic genotypes or anatoxin production remain unknown.

## Data Availability Statement

The datasets generated for this study can be found in NCBI SRA accession PRJNA578643.

## Author Contributions

SW, JP, and MH contributed to the conception and design of the study. SF, SW, JP, and KS acquired the samples and data. OL performed the statistical analysis. GT-L, OL, SF, and SW contributed to the analysis and interpretation of the data. GT-L and SW wrote the first draft of the manuscript. All authors contributed to the manuscript revision, read, and approved the submitted version.

## Conflict of Interest

The authors declare that the research was conducted in the absence of any commercial or financial relationships that could be construed as a potential conflict of interest.
