# Supplementary material for: Broad and Fine Scale Variability in Bacterial Diversity and Cyanotoxin Quotas in Benthic Cyanobacterial Mats
Source: Front Microbiol. 2020 Feb 6;11:129. doi: 10.3389/fmicb.2020.00129 (PMC7017413; doi:10.3389/fmicb.2020.00129)
Supplement: Supplementary file 1 [file Data_Sheet_1.docx]

**Supp. Material 1** Geographic coordinates for both Hutt and Cardrona sampling sites.

| **River** | **Site ID** | **Latitude** | **Longitude** |
| --- | --- | --- | --- |
| Hutt | 1 | -41.090689 | 175.097597 |
| Hutt | 2 | -41.111594 | 175.095076 |
| Hutt | 3 | -41.118839 | 175.041902 |
| Hutt | 4 | -41.145083 | 174.997987 |
| Hutt | 5 | -41.198274 | 174.917497 |
| Hutt | 6 | -41.204141 | 174.907233 |
| Cardrona | 1 | -44.725599 | 169.152347 |
| Cardrona | 2 | -44.719227 | 169.156569 |
| Cardrona | 3 | -44.718683 | 169.157931 |
| Cardrona | 4 | -44.711256 | 169.164263 |
| Cardrona | 5 | -44.699167 | 169.177222 |
| Cardrona | 6 | -44.685071 | 169.197192 |
