# Supplementary material for: Broad and Fine Scale Variability in Bacterial Diversity and Cyanotoxin Quotas in Benthic Cyanobacterial Mats
Source: Front Microbiol. 2020 Feb 6;11:129. doi: 10.3389/fmicb.2020.00129 (PMC7017413; doi:10.3389/fmicb.2020.00129)

Suppl. Material 2 Rarefaction curve showing the number of individual sequence units (ISU) versus the number of reads in each sample from the Hutt River (solid line) and Cardrona River (dashed line). Sample sizes were rarefied to 2,500 reads (indicated by the red dotted line) for beta diversity analysis. The x-axis is square-root transformed.

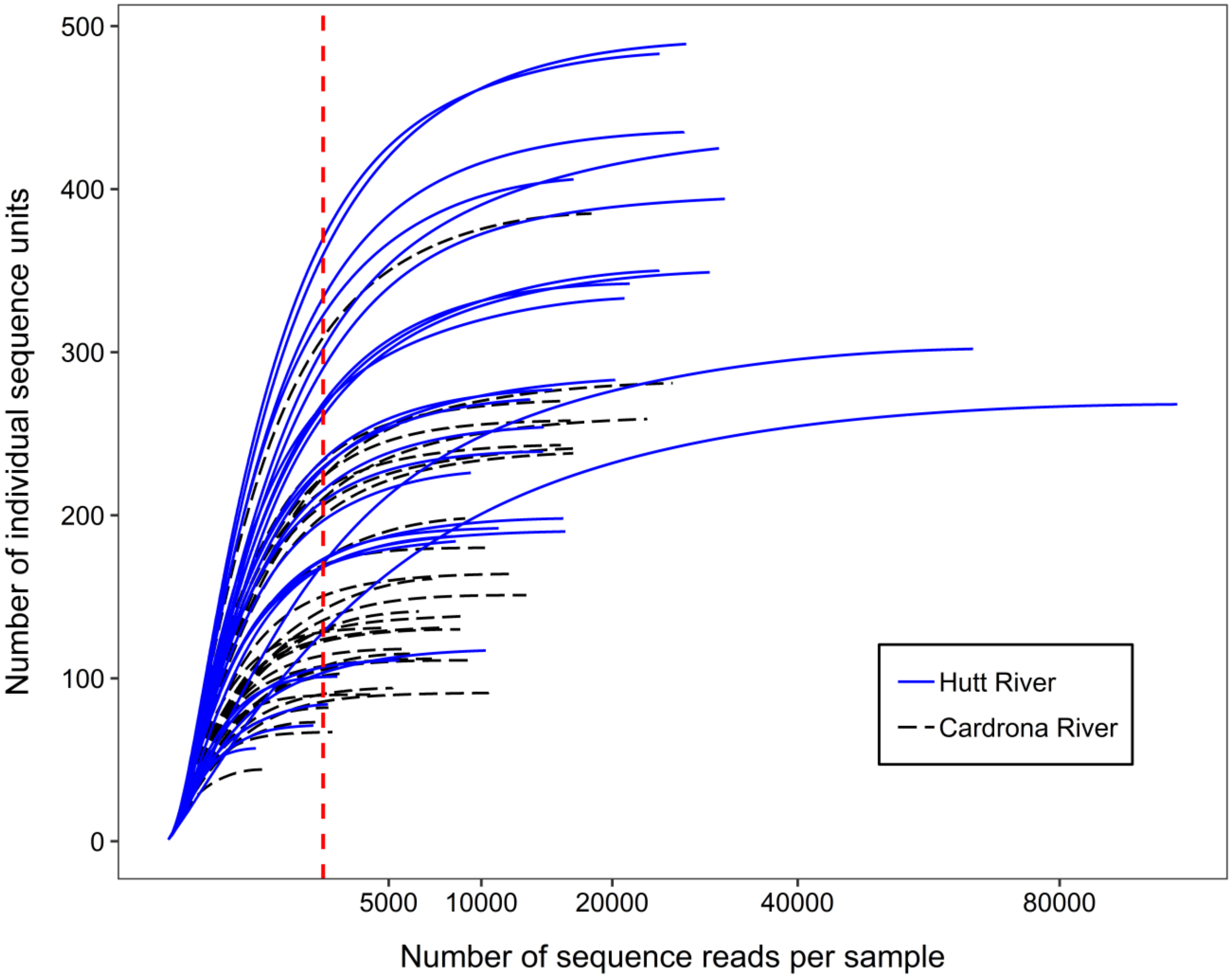

Supplement: Supplementary file 2 [file Data_Sheet_2.pdf]
