# Supplementary material for: Broad and Fine Scale Variability in Bacterial Diversity and Cyanotoxin Quotas in Benthic Cyanobacterial Mats
Source: Front Microbiol. 2020 Feb 6;11:129. doi: 10.3389/fmicb.2020.00129 (PMC7017413; doi:10.3389/fmicb.2020.00129)

Suppl. Material 3 Single point measurements of: (A, B) *Microcoleus autumnalis* cover (%), (C, D) conductivity, (E, F) dissolved inorganic nitrogen (DIN), (G, H) dissolved reactive phosphorus (DRP), (I, J) temperature, and (K, L) water velocity at sites along the Hutt River, (Wellington; New Zealand) and the Cardrona River, (Otago; New Zealand). Sites are arranged along the x-axis from upstream (left) to downstream (right).

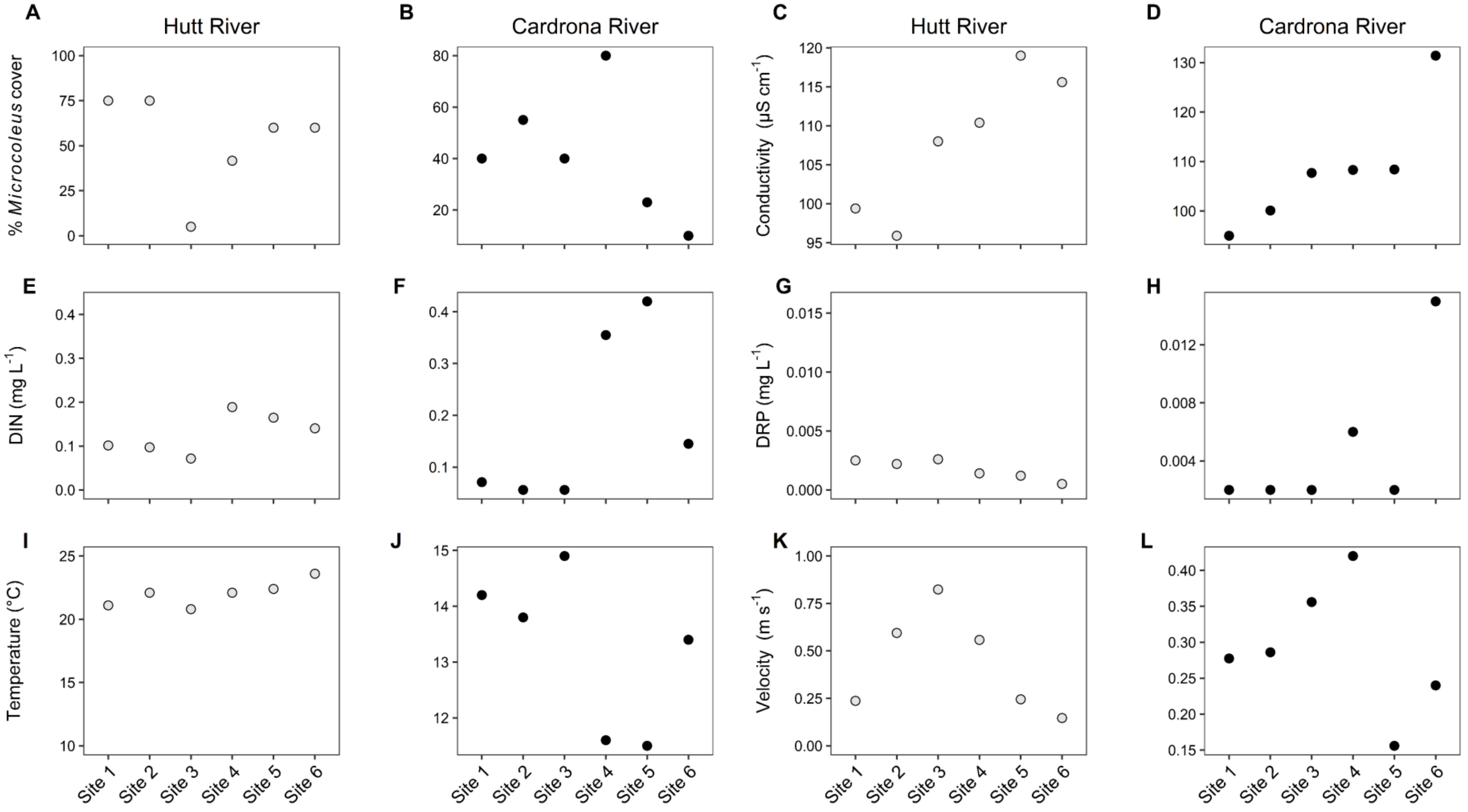

Supplement: Supplementary file 3 [file Data_Sheet_3.pdf]
