# Supplementary material for: Broad and Fine Scale Variability in Bacterial Diversity and Cyanotoxin Quotas in Benthic Cyanobacterial Mats
Source: Front Microbiol. 2020 Feb 6;11:129. doi: 10.3389/fmicb.2020.00129 (PMC7017413; doi:10.3389/fmicb.2020.00129)
Supplement: Supplementary file 4 [file Data_Sheet_4.docx]

**Suppl. Material 4** P-values from the pair-wise PERMANOVA analysis comparing bacterial communities among six sites in Hutt River, Wellington. Non-significant results (p > 0.05) are shown in bold.

| **Hutt River Site** | 1 | 2 | 3 | 4 | 5 | 6 |
| --- | --- | --- | --- | --- | --- | --- |
| 1 |  | 0.0098 | 0.0077 | 0.0075 | 0.0081 | 0.0077 |
| 2 |  |  | **0.1946** | 0.0262 | 0.0303 | 0.0082 |
| 3 |  |  |  | 0.0071 | 0.008 | 0.0099 |
| 4 |  |  |  |  | 0.03 | 0.0068 |
| 5 |  |  |  |  |  | 0.007 |
