# Supplementary material for: Broad and Fine Scale Variability in Bacterial Diversity and Cyanotoxin Quotas in Benthic Cyanobacterial Mats
Source: Front Microbiol. 2020 Feb 6;11:129. doi: 10.3389/fmicb.2020.00129 (PMC7017413; doi:10.3389/fmicb.2020.00129)
Supplement: Supplementary file 5 [file Data_Sheet_5.docx]

**Suppl. Material 5** P-values from the pair-wise PERMANOVA analysis comparing bacterial communities among six sites in Cardrona River, Otago.

| **Cardrona River Site** | 1 | 2 | 3 | 4 | 5 | 6 |
| --- | --- | --- | --- | --- | --- | --- |
| 1 |  | 0.0083 | 0.0073 | 0.0083 | 0.0097 | 0.0074 |
| 2 |  |  | 0.0074 | 0.0075 | 0.0293 | 0.0077 |
| 3 |  |  |  | 0.0086 | 0.0093 | 0.0074 |
| 4 |  |  |  |  | 0.0114 | 0.0081 |
| 5 |  |  |  |  |  | 0.0088 |
